# 758 Constant evolution: Early experiences treating COVID-19 in a burn center

**DOI:** 10.1093/jbcr/irac012.311

**Published:** 2022-03-23

**Authors:** Anthony P Basel, Garrett W Britton, Craig Yugawa, Leopoldo C Cancio

**Affiliations:** United States Army Institute of Surgical Research, Fort Sam Houston, Texas; US Army Institute of Surgical Research, JBSA Fort Sam Houston, Texas; Brooke Army Medical Center, Fort Sam Houston, Texas; US Army Institute of Surgical Research, JBSA Fort Sam Houston, Texas

## Abstract

**Introduction:**

The COVID-19 pandemic came as an unexpected challenge to many healthcare systems around the world. Many centers struggled to provide COVID-19 ICU-level care while also maintaining adequate care for non-COVID-19-related conditions, especially in critical care specialty units like trauma and burn. We present a case series of our early experiences treating COVID-19 in a burn center.

**Methods:**

We present a case.

**Results:**

See Table 1. Though one case was admitted prior to initiation of universal testing, routine infection-control protocols limited exposure to personnel and prevented transmission to staff. In May 2020, we implemented the use of N95 mask and eye protection during all aerosolizing procedures, N95 mask use in all ORs, and universal surgical mask use in all rooms regardless of COVID-19 status. An in-house risk-stratification system was used to screen patients based on symptoms and exposure. Burn-center admissions were screened at a lower threshold than throughout the institution given the unique nature of burn injury. Eventually, because of increasing community spread, all admissions to the hospital were universally screened with RT-PCR prior to admission. To minimize exposure to non-COVID patients and Burn Center staff, COVID-19 positive burn admissions were assessed on a case-by-case basis. High acuity patients were admitted to the Burn Center and followed by the COVID consult team. Lower acuity patients were admitted to the Burn Center but were treated on the medical COVID unit and followed by the burn consult service.

**Conclusions:**

The COVID 19 pandemic has strained healthcare systems worldwide. Development and implementation of universal screening, testing, infection-control precautions, and triage strategies are critical elements of burn care during the COVID-19 pandemic. As we prepare for future surges due to more transmissible variants, implementation of standard protocols enables continued provision of quality care, preservation of the healthcare workforce, and efficient use of resources.

